# Ultrafast optical imaging techniques for exploring rapid neuronal dynamics

**DOI:** 10.1117/1.NPh.12.S1.S14608

**Published:** 2025-02-27

**Authors:** Tien Nhat Nguyen, Reham A. Shalaby, Eunbin Lee, Sang Seong Kim, Young Ro Kim, Seonghoon Kim, Hyunsoo Shawn Je, Hyuk-Sang Kwon, Euiheon Chung

**Affiliations:** a Gwangju Institute of Science and Technology, Department of Biomedical Science and Engineering, Gwangju, Republic of Korea; b Massachusetts General Hospital, Athinoula A. Martinos Center for Biomedical Imaging, Charlestown, Massachusetts United States; c Harvard Medical School, Department of Radiology, Boston, Massachusetts, United States; d Tsinghua University, Institute for Brain and Cognitive Sciences, Beijing, China; e Hangzhou Zhuoxi Institute of Brain and Intelligence, Hangzhou, China; f Duke-NUS Medical School, Program in Neuroscience and Behavioral Disorders, Singapore; g Gwangju Institute of Science and Technology, AI Graduate School, Gwangju, Republic of Korea

**Keywords:** ultrafast optical imaging, voltage imaging, kilohertz two-photon microscopy, light field microscopy, event-based imaging, neuroimaging

## Abstract

Optical neuroimaging has significantly advanced our understanding of brain function, particularly through techniques such as two-photon microscopy, which captures three-dimensional brain structures with sub-cellular resolution. However, traditional methods struggle to record fast, complex neuronal interactions in real time, which are crucial for understanding brain networks and developing treatments for neurological diseases such as Alzheimer’s, Parkinson’s, and chronic pain. Recent advancements in ultrafast imaging technologies, including kilohertz two-photon microscopy, light field microscopy, and event-based imaging, are pushing the boundaries of temporal resolution in neuroimaging. These techniques enable the capture of rapid neural events with unprecedented speed and detail. This review examines the principles, applications, and limitations of these technologies, highlighting their potential to revolutionize neuroimaging and improve the diagnose and treatment of neurological disorders. Despite challenges such as photodamage risks and spatial resolution trade-offs, integrating these approaches promises to enhance our understanding of brain function and drive future breakthroughs in neuroscience and medicine. Continued interdisciplinary collaboration is essential to fully leverage these innovations for advancements in both basic and clinical neuroscience.

## 1 Introduction

Understanding neuronal activity is essential for studying brain function and disease, especially in the context of neurodegenerative disorders such as Alzheimer’s,^1^^–^^3^ Parkinson’s,^4^^–^^6^ and Huntington’s,^7^^–^^9^ as well as conditions such as neuropathic pain^10^^–^^12^ and chronic pain.^13^^–^^15^ Advances in neuroimaging, particularly in volumetric imaging techniques, have significantly enhanced our ability to explore these areas. Unlike traditional methods that provide only planar views, volumetric imaging provides a three-dimensional perspective, capturing depth information that has revolutionized our understanding of brain function and pathology. These technologies have enabled unprecedented spatial and temporal resolution, marking a major leap forward in the study of the brain.

**Fig. 1:**
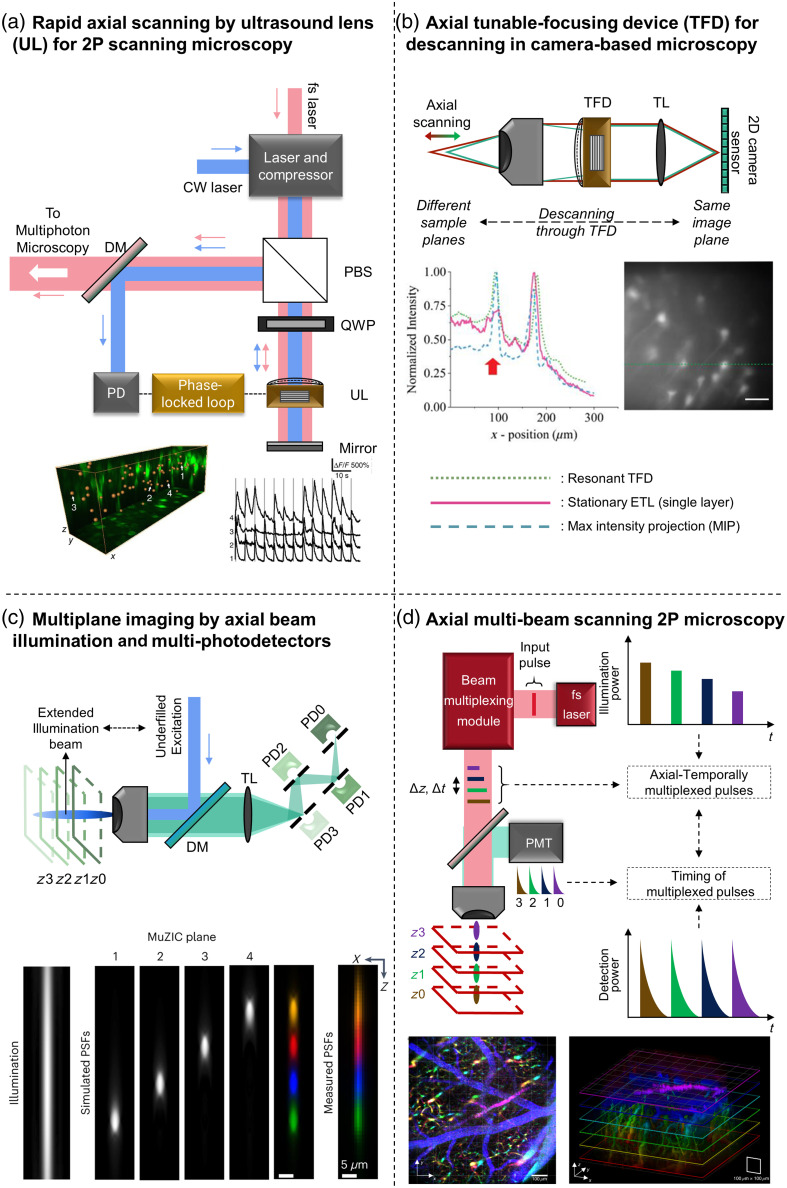
Examples of neuro-volumetric imaging (a) 3D view of recorded calcium signals in mouse brain with optical axial scanning by phase locked mechanism for ultrasound lens in two-photon microscopy to control it with better precision to reduce distortion. (b) ETL-descanning method brought the information at the different sample planes to the same image plane on camera. Comparison of intensity profiles of 80-μm-thick Thy1-eYFP mouse brain tissue slide images obtained via fixed, resonant, and stationary ETL modes. Scale bar: 50  μm. (c) A volumetric confocal method with multiple z-capturing simultaneously with multi-photodetectors for optical sectioning imaged the spontaneous activity. The corresponding simulated PSF for MuZIC (multi-z imaging with confocal detection) in different layers, as shown below; scale bar: 5  μm. (d) Deep imaging with two-photon excitation based on a reverberation loop. The plane separation Δz is dependent on the time delayed Δt between the multiplexed pulses. PD, photodiode; PBS, polarizing beam splitter; QWP, quarter waveplate; fs laser, femto-second laser; CW laser, continuous-wave laser; ETL, electrical tunable lens; TL, tube lens; DM, dichroic mirror; PMT, photomultipliers. The image of neurons and graph in panel (a) is reproduced with permission from Ref. 20. The image of neurons and graph in panel (b) is adapted with permission from Ref. 21. MuZIC plane simulation is reproduced with the permission from Ref. 22. The images of neurons are reproduced with permission respectively from Ref. 23.

In pre-clinical studies, the evolution of imaging techniques from wide-field microscopy (WFM) to confocal, light sheet, and multiphoton microscopy has significantly advanced volumetric visualization, a topic discussed further in Sec. 3.1. These developments have led to rapid 3D volumetric observation, overcoming the limitations of traditional methods such as z-translation stages, which, although effective at capturing depth, were relatively slow.^16^ To address the need for speed, microscopes have been developed with fast axial scanning capabilities and have been developed using mechanical devices such as electrically tunable lenses (ETL),^17^ adaptive lenses,^18^ spatial light modulators,^19^ or tunable acoustic gradient lenses (or ultrasound lens—TAG lens). For example, a phase-locked TAG lens [Fig. 1(a)] has been used to perform 10-Hz calcium volumetric multiphoton microscopy in mice.^20^ However, this system has limited depth scanning capability due to image distortion caused by the ultrasound lens. Other advances include hybrid methods that combine lateral scanning such as^24^^–^^26^ with axial scanning such as ETL and random-access scanning for neuro-tracking in different depths. This technique has been developed to perform sheet scanning or volume scanning (e.g., ETL-based plane-scanning^21^ or swept confocally-aligned planar excitation—SCAPE microscopy^27^). Especially, the ETL-quipped microscope illuminates excitation lights on different layers, thanks to “ETL-descanning;” the signal is collected on the same camera detector plane without further resampling [Fig. 1(b)].^21^

Another method of depth extension is the simultaneous imaging of multiplanes. Multi-z confocal microscopes using prisms,^28^ pinholes,^29^ or slits^30^ have further improved volumetric acquisition rates. An illustration of this technique is provided by Weber et al.^22^ with a demonstration of voltage imaging for *in vivo* neuroimaging [Fig. 1(c)]. Two-photon or multiphoton microscopy enables intravital optical imaging with submicron spatial resolution and tissue penetration depths of 0.5 to 1.5 mm. Incorporating a reverberation loop inserted into the laser pulse has enhanced the depth-capture capability of two-photon imaging,^23^ allowing for large fields of view and video-rate speed [Fig. 1(d)]. The system generates a series of foci from each input pulse independently, thereby preserving the axial resolution, which may otherwise be compromised in other fast z-scanning techniques, such as those using a TAG lens. This helps image various cell types in different depths while maintaining resolution [Fig. 1(d)]. A comprehensive review of volumetric imaging can be found in Mertz.^31^ Despite these advances, these techniques are limited by imaging speed, achieving only video rate and low temporal resolution. Given that typical neuronal firing rates range from 0.1 Hz in cortical neurons up to 500 Hz in fast-spiking interneurons, the complex dynamics of the brain require imaging techniques capable of capturing ultrafast cellular and molecular processes in real-time.

Ultrafast imaging can be defined in various ways, including the ability to record fast motion without blurring,^32^ the application of serial ultra-short pulses,^33^ to achieve real-time imaging.^34^ However, in this review, “ultrafast” specifically refers to the potential of kHz imaging and high temporal resolution. Ultrafast optical imaging technologies have broad applications in neurobiological and clinical research, playing an important role in drug testing, understanding neurological diseases, and developing new treatment strategies. These technologies provide detailed insight into brain structure and function, paving the way for breakthroughs in neuroscience and medicine.

In volumetric imaging, achieving video-rate speed may not be sufficient for neuroimaging. The main factors contributing to this limitation include:

1. Data acquisition: volumetric visualization demands significant time to acquire multiple slices or z-stacks.
2. Hardware limitations: typical camera frame rates are generally limited to several 100 frames per second (fps) at full frame, which may not suffice for ultrafast imaging.
3. Spatial resolution trade-off: increasing fps to achieve high temporal resolution often compromises spatial resolution, affecting the clarity of image details.

This review will explore the latest and potential strategies to overcome these limitations in volumetric imaging, with a focus on their application in the field of neuroscience.

## 2 Need for Speed and Sensitivity: Real-Time Neuronal Activity Indicators for Calcium and Membrane Potentials

Understanding the electrical signals or membrane potential changes of neurons is essential for identifying and investigating potential brain diseases. Traditional methods have used electrodes to monitor neuronal activity^35^ and have provided valuable insights but have faced limitations in fully capturing neuronal activity. This limitation highlights the growing appeal of direct fluorescence imaging of voltage as an attractive and complementary approach for studying neural circuits. Alongside these traditional electrode-based techniques, live-cell ${\mathrm{Ca}}^{2+}$-based imaging has significantly advanced our understanding of cellular communication, especially in neurons. Numerous studies have investigated the role of calcium influx in both presynaptic and postsynaptic regions,^36^^,^^37^ as well as in gene transcription^38^^,^^39^ and other cellular processes. By monitoring calcium dynamics, researchers can indirectly track neuronal activity, decode signaling pathways, and gain deeper insights into synaptic plasticity and overall brain function.

Standard measurements of ${\mathrm{Ca}}^{2+}$ signals typically rely on genetically encoded calcium indicators with high sensitivity, such as the popular GCaMP family, including advanced sensors such as GCaMP5,^40^ GCaMP6,^41^ jGCaMP7,^42^ and jGCaMP8.^43^ Real-time ${\mathrm{Ca}}^{2+}$ tracking through multiphoton microscopy of live brain tissue has proven to be a powerful tool for assessing the impact of neurotoxicants on brain function, providing valuable insights into the mechanisms of neurotoxicity and its broader implications for human health.^44^ In neurodegenerative disorders such as Alzheimer’s disease (AD) and Parkinson’s (PD) disease, impaired mitochondrial ${\mathrm{Ca}}^{2+}$ regulation leads to the production of excessive reactive oxygen species (ROS), triggering cell death, and accelerated disease progression.^45^ However, calcium imaging has its limitations. First, calcium imaging indicators have slow response times,^46^ even though some research has been conducted on spike inference using deep learning–based methods^47^^,^^48^ to improve temporal resolution.^49^ Second, they only indirectly reflect action potentials.^46^^,^^50^ Although they excel at providing spatial maps of neural activity at specific time points, they may not fully capture the dynamic interplay of information within complex neural circuits.^51^

By measuring subthreshold voltage dynamics from synaptic or ion channels,^52^ researchers can gain critical insights into neuronal circuits. Voltage imaging presents a promising solution as it directly measures changes in membrane potential, offering faster and more precise recordings of neuronal activity than calcium imaging. To accurately capture action potential profiles, especially for fast-spiking interneurons, voltage imaging requires frame rates exceeding 1000 fps—approximately 100 times faster than traditional calcium imaging.^52^

This technique employs voltage-sensitive dyes (VSD) or genetically encoded voltage indicators (GEVIs) to directly measure membrane potential changes. VSD imaging has been explored as a method to visualize large populations of neurons with high resolution, typically ranging from 20 to 50  μm, and temporal resolutions in the millisecond range.^53^ These dyes bind to the cell membrane of the targeted samples and convert the electrical signal of the potential into an emitted light signal. Initially developed ∼40 years ago, VSDs were successfully applied to animal models, including rodents, ferrets, and salamanders.^54^^–^^59^ As the technology advanced, more *in vivo* studies emerged in the early 2000s, further validating the technique.^60^^–^^63^ For a comprehensive history of VSD imaging, see the review by Chemla and Chavane.^64^

VSDs offer advantages in resolution and broad applicability; however, there are limitations related to phototoxicity and photobleaching, which restrict their effectiveness in long-term studies. In addition, VSDs can result in non-specific labeling because they display the same behavior across all cellular compartments, without distinction other than depth.^35^ GEVIs, introduced around 30 years ago, address many of these limitations. They enable the targeting of specific cell types or compartments using genetic promoters or organelle-specific localization peptides, thereby enhancing the precision in voltage signal localization within designated targets. Moreover, GEVIs typically cause less phototoxicity than VSDs, making them more suitable for long-term use. According to Ref. 65, there are three main families of GEVIs: voltage-sensitive domain-based GEVIs (VSD-based GEVIs), Opsin-based GEVIs, and Chemigenetic indicators.

The first family of GEVIs is based on a VSD coupled with a fluorescent protein. The earliest of these, FlaSh, was introduced in 1997,^66^ but it had limited applications in mammalian systems. Subsequent advancements, such as ArcLight,^67^^–^^69^ accelerated sensor of action potentials (ASAP),^70^^–^^72^ improved performance, and broadened applicability, particularly for two-photon microscopy (2PM). Although most indicators in this family are effective within the cyan or yellow fluorescent protein range, direct applications for 2PM have been limited. However, new indicators such as Marina,^73^ FlicR,^74^^,^^75^ and Ilmol^76^ are currently under investigation, pushing the boundaries of what these tools can achieve. In 2023, SpikeyGi and SpikeyGi2 were developed as GEVIs with a positive fluorescence–voltage slope relationship, successfully applied for kilohertz scanning using 2PM.^77^ This advancement marked a significant step forward as this first family has demonstrated wide-range applications for neuron recording both *in vitro* and *in vivo*, as shown in previous studies.^78^^–^^82^ However, challenges such as low signal-to-noise ratios (SNRs) and photobleaching still persist. Within this family, it is notable that the ASAP indicators stand out, with ASAP4,^83^ JEDI2P,^84^ and ASAP6.^85^

The second family of GEVIs operates primarily within an emission wavelength range of ∼515 to 715 nm, with rhodopsin serving both as a fluorescent reporter and a voltage sensor. Another class within this family employs rhodopsin linked to a fluorescent tag, functioning as a VSD, through electrochromic Förster resonance energy transfer (eFRET) mechanism^86^ or fluorescent protein–retinal fluorescence resonance energy transfer (FRET).^65^ The first opsin-based GEVI, proteorhodopsin optical proton sensor (PROPS) was introduced in 2011.^87^ However, it faced limitations in applicability to mammalian neurons, leading to the development of the Arch indicator in the following year.^88^ Although Arch addressed some of the shortcomings of PROPS, it struggled with a low SNR. In response, subsequent indicators such as QuasAr types,^89^^,^^90^ Archon,^91^ and Ace2-4aa-mNeon^92^ were developed, each offering improvement in SNR. Some older opsin-based GEVIs were not suitable for two-photon imaging techniques. To address this limitation, multi-z imaging with confocal detection (MuZIC) was applied using the indicator GEVI Voltron2-ST^93^ to image mouse motor cortex neurons.^22^ In addition, an opsin-based GEVI called Jarvis, which incorporated with AaFP1, the brightest known fluorophore, was recently compared with the pAce and JEDI-2P indicator for two-photon voltage imaging, highlighting the ongoing evolution and potential of these tools.^94^

Despite ongoing advancements, GEVIs still face challenges such as low brightness and slow kinetics, limiting their overall utility. To address these issues, a new class of indicators known as “chemigenetic indicator” has emerged, combining small molecule fluorophores and genetic components^95^^–^^97^ The first of these, hybrid voltage sensor (hVOS),^98^ marked the beginning of FRET chemigenetic sensors. This innovation paved the way for more advanced chemigenetic probes such as VF-EX,^99^ which incorporates an enzyme to enhance performance. In addition, other chemigenetic probes such as VoltageSpy,^100^ STeVI1,^101^ and Solaris^102^ have been developed, each encoded with protein tags to improve *in vivo* applicability. These advances represent a significant step forward in voltage imaging, offering potential solutions to the limitations of earlier GEVIs. Table 1 provides a brief summary of some of the most advanced GEVIs currently available, highlighting their contributions to the field.

**Table 1:** Example of some latest GEVIs.

Type	Indicator	Example	Excitation—emission (nm)	ΔF/F for 100 mV	Key features and limitations	Reference
VSD-based GEVI	Monochromatic-FP	FlicR1	561 to 630	∼6.4%	Sensitive to voltage changes but issues with photostability and localization	75
		FlicR2	561 to 530	∼13%	Improved ΔF/F sensitivity with twice the sensitivity of FlicR1 but still has photostability and localization issues	75
		ASAP4b	480 to 520	180%	Enhanced photostability, suitable for one- and two-photon microscopy	83
		ASAP4e	480 to 520	210%	Similar to ASAP4b, optimized for extended recordings	83
		SpikeyGi	470 (1P)/920(2P)—525	∼19%	High sensitivity, robust for *in vitro*	77
		SpikeyGi2	470 (1P)/920(2P)—525	∼58%	Higher sensitivity than SpikeyGi, robust for *in vitro* and *in vivo*, potential for higher background fluorescence	77
		JEDI-2P (2024)	470(1P)/940(2P)—green	(1P) ∼46%	High contrast and SNR, slower kinetics compared with Jarvis and pAce	94
				(2P)---^a^		
		ASAP6.1	470(1P)/960(2P)—green	204%	Higher sensitivity than ASAP3, faster activation kinetics than ASAP4, better in relative response per AP	85
		ASAP6.2	470(1P)/960(2P)—green	161%	Higher sensitivity than ASAP3, faster activation kinetics than ASAP4, better in per-molecule SNR for Aps in a brighter range	85
	Dual-FP	VSFP Butterfly	483/542 (donor)—594 (acceptor)	∼22%	Reduce fluorescent protein aggregation and background noise compare to previous version VSFP2.42	103
Opsin-based GEVI	FP-retinal FRET	VARNAM	558 to 605	−14% (120 mV)	High brightness and kinetics, uncertainty for multiphoton imaging	75
		Jarvis	470(1P)/940(2P)—green	(1P) ∼29%	High-brightness and narrow spectrum reduces crosstalk; sensitivity reduces at high irradiances	94
				(2P)---^b^		
		pAce	470(1P)/940(2P)—green	(1P) ∼41%	Broaden spectrum allows wide-range excitation but increases crosstalk	94
				(2P)---^b^		
	Rhodopsin-based	Archon1	635-NIR	∼43%	Good membrane localization, brightness, and sensitivity improvement require higher excitation intensity	91
		Archon2	635-NIR	∼19%	Faster kinetics and lower voltage sensitivity but lower SNR than Archon1	91
Chemigenetic GEVI	FP-dye FRET	hVOS	480 to 535	34%	Large dynamic range, fast recording, consideration of phototoxicity	98, 104
	Photo-induced electron transfer	VoltageSpy	475 to 540	∼60%	Higher sensitivity of some common GEVIs	100
			542 to 650			
	Opsin-dye FRET	FlareFRET (Flare1)	488 to 525	∼36%	High sensitivity, wide dynamic range	105
		STeVI1	∼550 to ∼600	∼5.5%	Wash-free imaging, less power, high speed	101
		Voltron2	561 to 570	∼55%	Improve the quality of the mouse brain images	93
		Voltron2-ST	595 to 650	∼45%	Faster response time, higher sensitivity, and targeting accuracy than Voltron2	93
		${\text{Solaris}}_{585}$	488	∼61%	More sensitivity and faster response than Voltron2	102

FP, fluorescent protein.

“---” No information.

a Constant across a range of irradiances.

b Reduced significantly at high irradiance; optimal at low irradiance.

With its valuable features, voltage imaging is becoming a powerful tool in neuroimaging. Variant types of voltage indicators open multiple options for application, so the appropriate choice should depend on the characteristics of the indicator, such as dynamic range and response time. Voltage imaging is a promising tool for understanding neuronal function and dynamics. This tool offers high temporal resolution with VSDs and long-term use with more specific targets by GEVIs. This technique provided several applications. For example, Walker et al.^106^ used VSDs to map neuronal activity in cultured hippocampal neurons arranged in patterned microislands with both healthy and diseased states. Targeted illumination strategies further enhanced GEVI voltage imaging by improving signal contrast and reducing noise, leading to more accurate observations of brain function and aiding in the study of neurological diseases.^107^ Another study utilized GEVIs and optogenetic activation, a specific subpopulation of V3 neurons control swim strength and duration without influencing tail beat frequency. These neurons are essential for enhancing motor output; optogenetic activation increased swimming vigor, whereas their ablation impaired the zebrafish’s ability to adapt their swimming behavior.^108^

Voltage imaging still faces challenges related to significant noise and limitations in illumination techniques. Differences in illumination colors can lead to varying levels of scattering and tissue absorbance. Blue light, commonly used in scientific and biomedical applications such as optogenetics and imaging, has high photon energy, and excessive or prolonged exposure can cause unintended inhibitory effects on biological processes.^109^ Phototoxicity can be mitigated using longer wavelengths with lower photon energy, such as near-infrared light in two-photon imaging. However, the choice of indicator must align with the illumination method. For instance, JEDI-1P performs exceptionally well in single-photon microscopy due to its superior brightness, photostability, and speed, compared with JEDI-2P, which is specifically optimized for two-photon imaging. By contrast, JEDI-1P is more prone to photolability under two-photon illumination conditions.^110^ GEVIs rely on cellular protein trafficking through the endoplasmic reticulum and Golgi apparatus to localize to the membrane. However, as most GEVIs are derived from transgenic sequences, their membrane targeting is often inefficient, necessitating substantial optimization efforts.^111^ To enhance labeling, strategies include the use of export sequences (e.g., from the endoplasmic reticulum or Golgi) derived from voltage-gated ion channels^109^ or improving the coupling between VSD/opsin and fluorescent proteins.^112^ Targeting efficiency may vary significantly between experimental conditions, such as *in vivo* and *in vitro* setups.

Deep learning has become a worthwhile tool for advancing SNR by denoising.^113^^,^^114^ Mostly applied to voltage imaging is self-supervised deep learning as supervised-based methods require clear and labeled data, which may not be practical for voltage imaging. The available self-supervised methods over the past 5 years for denoising neuron data include Noise2Void in 2019,^115^ DeepInterpolation in 2021,^116^ DeepCAD in 2021,^117^ DeepVID in 2023,^77^ and SUPPORT.^118^ So far, deep learning–based approaches for voltage imaging have been developed, such as CellMincer^119^ and DeepVID v2.^120^ All of them significantly optimize voltage data processing and efficiently improve neuron depiction.

## 3 Imaging Strategies for Neurosciences: from Video Rate to Ultrafast

As mentioned in Sec. 1, voltage imaging represents a promising approach for investigating neuronal interactions as it demands high-speed imaging techniques to accurately capture dynamic membrane potential changes. Although conventional imaging techniques such as WFM, confocal microscopy, and 2PM provide detailed cellular-level observations, they are often constrained by their limited imaging speed. Rapid imaging is crucial not only for capturing the fast pace of neuronal firing but also for minimizing potential cellular damage caused by intense illumination. Therefore, Secs. 3.1–3.3 will explore three strategies that enable ultrafast processing, which are essential for advancing our understanding of neuronal dynamics.

### 3.1 Kilohertz Scanning-Based Strategy: from Sub-kilohertz to Over-Kilohertz

WFM is a well-established technique for visualizing biological samples. It works by illuminating the entire sample using light sources, typically lamps or LEDs, and then magnifying the information with an objective lens before projecting it onto an imaging sensor, such as a camera. Although WFM has a simple design with a large field of view (LFOV), it is limited in providing depth detail for thick samples and has a diffraction-limited resolution.^121^ Confocal microscopy (CM), on the other hand, improves image clarity using a pinhole aperture to eliminate out-of-focus light, thereby reducing image blurring from regions outside the focal plane.^122^ This makes CM a good example of optical sectioning for 3D imaging,^123^ though it is limited by penetration depth due to sample scattering and is prone to photobleaching.

To address photobleaching and enhance imaging speed, light sheet microscopy (LSM) was employed, enabling *in vivo*^124^^,^^125^ and *in vitro*^126^^,^^127^ structures. Conventional LSM separates the emission and excitation paths and uses a thin sheet of light to illuminate samples instead of point-by-point scanning.^128^ Although LSM offers significant benefits in reducing photobleaching and improving penetration depth compared with CM, it also has drawbacks, including system complexity and challenges with imaging non-transparent samples.

Unlike traditional single-photon microscopy, which relies on single photons to excite fluorophores, multi-photon microscopy (MPM) is a powerful imaging technique that utilizes multiple photons for excitation.^129^ MPM, particularly in the form of 2PM, offers several advantages over single-photon methods. Using lower-energy photons, typically near-infrared light, 2PM reduces phototoxicity and allows for deeper tissue penetration.^130^ These properties make MPM especially valuable for live-cell imaging and visualization of thick tissue samples. However, the requirement for pulsed lasers and tight focusing in MPM can result in slower image acquisition times compared with some other techniques.

Traditional MPMs operate at frame rates of tens of frames per second, which is insufficient for capturing fast physiological events such as neuronal action potentials or calcium signals, which occur on the order of milliseconds. To overcome these limitations, sub-kilohertz solutions were optimized with fast axial scanning with variable focusing control devices such as TAG lenses^20^ or ETL,^131^ achieving an imaging speed of less than 100 Hz. In addition, 2.5D imaging techniques (i.e., 2D projection of 3D volume), such as those using Bessel beams,^132^^,^^133^ have been explored to improve imaging speed and temporal resolution.

**Fig. 2:**
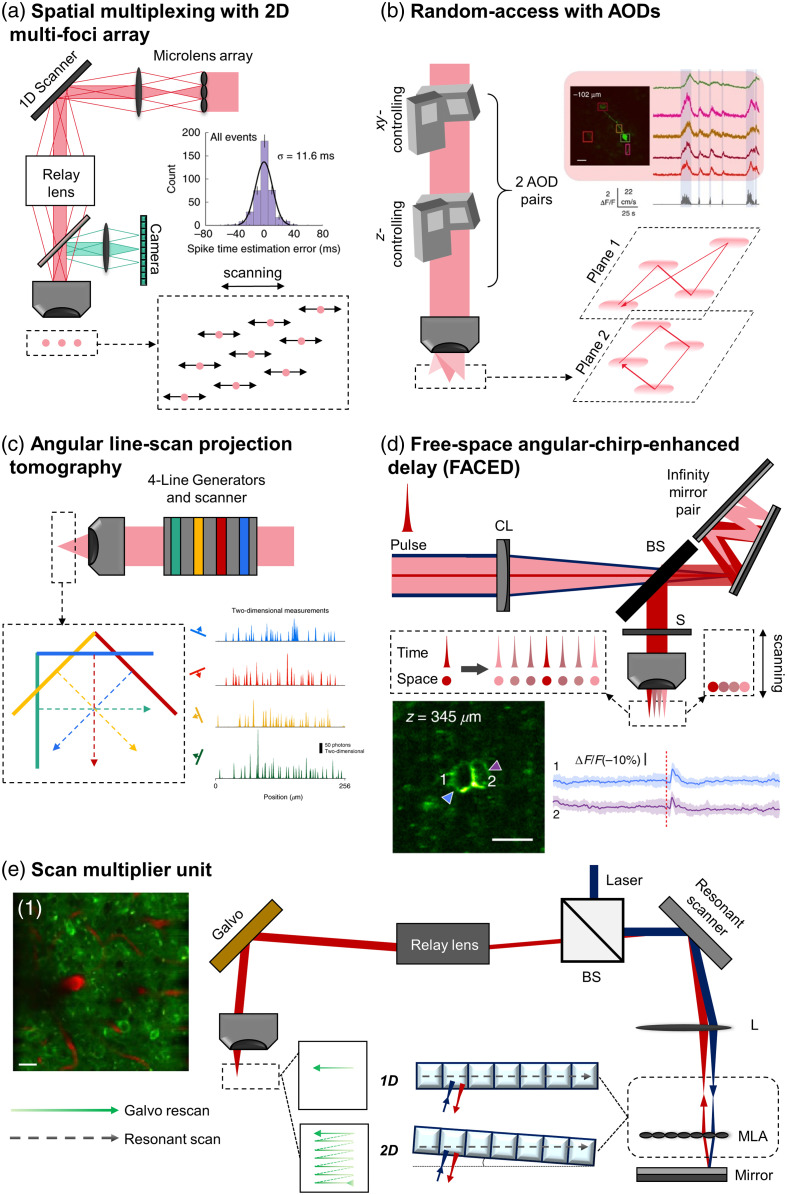
kHz-2PM principles with neurological. (a) Grid-foci scanning: a microlens array generates uniform beamlets for multiple foci on the sample plane, scanned by a 1D scanning mirror to accurately estimate neural spike timing, as shown in the accompanying histogram of spike timing errors. (b) Random-access imaging using acousto-optic deflectors (AODs): utilizes sound waves to quickly modulate laser deflection, enabling fast and precise imaging of specific neural regions. Image of neurons (scale bar: 20  μm) from Ref. 135 with fluorescent traces, showing speed (vertical scale bar) and time (horizontal scale bar). (c) Scanned line angular projection tomography: employs four simultaneous line scans to achieve high-resolution random-access imaging from different angles with two-dimensional measurements, beneficial for capturing dynamic neural activities. (d) Free-space angular-chirp-enhanced delay (FACED): uses a pair of mirrors and a cylindrical lens for time-stretch imaging, enabling high-speed imaging of neuronal activity with enhanced temporal resolution. Image of neurons from Ref. 136, with subthreshold ΔF/F, scale bar: 20  μm e) scan multiplier unit (SMU): combines an 8-kHz resonant scanner with a 1-kHz galvo scanner and a lenslet array, allowing flexible scanning patterns for comprehensive neural imaging. The resulting images show a composite of GCaMP (green) and Texas Red (red) signals, scale bar: 20  μm. MLA, microlens array; L, lens; OBJ, objective lens; CL, cylindrical lens; S, scanner; BS, beam splitter; AOD, acousto-optics deflector. The graph in panel (a) is reproduced with permission from Ref. 134; image of neurons in panel (b) is reproduced with permission from Ref. 135; the measurement in panel (c) is reproduced with permission from Ref. 137; image of neurons and analysis in panel (d) is reproduced with permission from Ref. 136; image of neurons in panel (e) is adapted with permission from Ref. 138.

To address the need for ultrafast tracking, kilohertz two-photon microscopy (kHz-2PM) was developed, achieving frame rates of up to several 1000 fps. This advancement allows for real-time imaging of rapid biological processes with high temporal precision. A common method for speed acceleration involves the use of scanning mirrors. For example, a study^134^ reported a technique combining a fast scanner with a tilted microlens array for grid-foci scanning, providing 1-kHz video imaging of calcium signals in awake mice at a depth of 300  μm [Fig. 2(a)]. However, this method introduced scattered fluorescence, contributing to background noise.

Several studies have combined 2PM with random-access imaging, mostly by acousto-optic deflector (AOD),^135^^,^^139^^–^^143^ which control light beams through sound waves, causing variations in the refractive index and acting like a diffraction grating. By inserting two AOD pairs for spatial scanning and depth control, these systems can access targeted regions and generally achieve 1-kHz planar recording, demonstrating the potential for neuron imaging through the random-access method [Fig. 2(b); example results from Ref. 135]. Notably, one study^144^ reported achieving up to 10 kHz.

Tomographic techniques that combine projection microscopy, which uses multiple measurements from different angles to reconstruct a 3D image, and random-access imaging, such as scanned line angular projection microscopy (SLAP),^137^ operate with four 200-nm spacing line scans, achieving an imaging speed of ∼2  kHz. This technique was recorded with higher accuracy than raster scanning in dendritic measurement [Fig. 2(c)].

A spectro-temporal encoding MPM, such as spectro-temporal laser imaging by diffracted excitation (SLIDE),^145^ uses inertia-free laser sweeping combined with diffraction grating, offering higher average powers compared with traditional 2PM without causing damage to samples. Free-space angular-chirp-enhanced delay (FACED)^146^ is a technique that utilizes a pair of parallel mirrors to manipulate light pulses in free space enabling time-stretched imaging. Introducing FACED [Fig. 2(d)]^136^ resulted in planar scanning within the range of 1 to 3 kHz, achieving subcellular resolution neural activity imaging in mouse brains. FACED-2PFM (FACED two-photon fluorescence microscopy)^147^ demonstrated 1-MHz line scanning and 1-kHz recording at depths of ∼800  μm in head-fixed awake mice. Besides its ultrafast imaging speed, the dual-beam excitation with adaptive excitation polygon-scanning multiphoton microscope^148^ provides an LFOV and deep imaging for neuronal recording with high spatial and temporal resolution in three-photon (3P) imaging, without sacrificing resolution to achieve higher imaging throughput. The main concept of this technique involves using a polygon scanner for large angle scanning at a speed of ∼11-kHz line rate, combined with two illumination modes: 2P with remote focusing control and 3P with a looped cavity design.

Currently, most of the above techniques can image only a few cells and require complex systems.^138^ Therefore, scan multiplier unit 2PM was introduced using a 16 by 1 lenslet array with an 8-kHz resonant scanner. When the microlens array is adjusted on the scanning axis, after the scanning by the resonant scanner, the light will be reflected to form a descanning on the resonant scanner before coming to the galvo scanner for rescanning, and then, the system will perform ultrafast 1D scanning on the sample. By rotating the microlens array by a suitable angle, the rescanning pattern can cover the FOV in 2D. This setup performed planar scanning at 1 kHz with 256 line-scans/second, imaging blood flow, neurovascular dynamics, and neuronal activity in deep tissue samples at kHz rates [Fig. 2(e)]. In addition, some kHz-2PM reports have illustrated their ability to perform voltage imaging, such as^142^ with GEVI ASAP2s and^136^^,^^141^^,^^143^ with GEVI ASAP3. Table 2 provides a summary of the above techniques and their recorded applications.

**Table 2:** Comparison of kHz-2PM techniques between their working principle, the imaging speed, and their application.

General principle	Technique specialty	Speed (Hz)	Application	Reference
Grid-foci scanning	1D scanner and titled microlens array	∼1000	Blood flow, neural activity, calcium imaging in awake mice	134
Random-access	4-projection-line-scans tomography	∼1000	Cells, neurons, volumetric calcium imaging, voltage imaging (RhoVR indicator)	137
	Acousto-optic deflector (AOD)	∼1000	Calcium imaging neuron	135, 139, 140
			Voltage imaging neuron (ASAP2s indicator)	142
			Voltage imaging neuron (ASAP3 indicator)	141, 143
		≤10,000	Calcium imaging neuron, blood flow	144
Spectro-temporal encoding	Wavelength-swept laser and diffraction grating	∼2000	Cells flow cytometry	145
FACED	Combined an infinity mirror pair with a cylindrical lens	∼1000 to 3000	Calcium imaging, glutamate imaging, voltage imaging of awake mice (ASAP3 indicator)	136
		∼1000	Blood flow	147
Scan multiplier unit	Descanning resonant scanner and rescanning galvanometer, with lenslet array	∼1000	Blood flow, calcium imaging neuron.	138
Adaptive excitation and beamlet scanning	Adaptive excitation, polygon scanning, and multi-focus	∼4000 to 11,000	Calcium imaging neuron	148

Traditional 2PM has been used to explore signal transduction mechanisms, such as astrocytic calcium signaling in the live murine brain, particularly during epileptic events. This method has identified increases in ${\mathrm{Ca}}^{2+}$ linked to vasodilation during each ictal event at the seizure focus. In distant regions, ${\mathrm{Ca}}^{2+}$ rises correspond with vasoconstriction at the onset of seizures and vasodilation in the later stages. These findings could inform the selection of drugs for seizure treatment.^149^ By incorporating various optical components, kHz-2PM significantly advances the study of rapid biological processes, providing new perspectives on *in vivo* imaging, especially for neuronal activity. kHz-2PM surpasses traditional 2PM, in terms of speed and resolution, highlighting the potential for further applications. However, its complexity remains a limitation, hindering widespread adoption.

### 3.2 Light-Field-Based Strategy: from 3D Imaging to 4D Imaging

Volumetric imaging, which relies on scanning methods, presents challenges when studying dynamic cells or neurons. Photobleaching is a significant concern as scanning often requires longer exposure times, especially when using fluorescent probes. Scanning methods, whether line-scan or point-scan, can also introduce visible artifacts into the final image. To address these issues, single-shot or scanless imaging techniques have been developed, though both scanning and scanless methods have limitations in capturing angular information. Angular information is crucial because objects are viewed in orthographic projection, making it challenging to distinguish overlapping structures.^150^

As mentioned previously, volumetric imaging is restricted by speed and depth recording limitations,^151^ highlighting the need for a single-shot 3D imaging method. To overcome these challenges, a technique that captures the “light field” known as light field microscopy (LFM) was introduced.^150^ LFM addresses the limitations of traditional methods by enabling 4D, capturing 2D in planar position (spatial) and 2D in angle (angular).

**Fig. 3:**
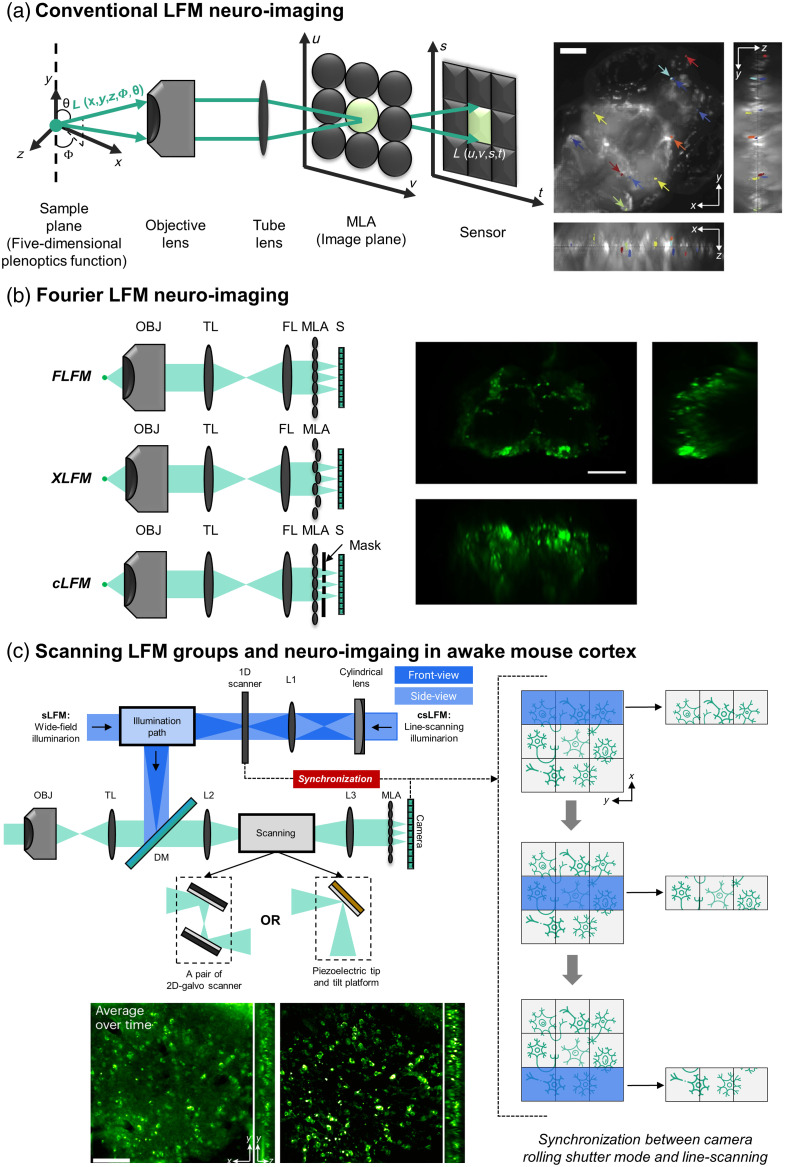
Configurations of LFM emission types: (a) five-dimensional plenoptic function L(x,y,z,Φ,θ) representation of light (green arrow) in space. The five-dimensional plenoptic function can be parameterized into a 4D representation L(u,v,s,t) called “light slab.” MLA is placed at the image plane of the LFM to capture the 4D information (u,v,s,t) and perform conventional LFM and performed in zebrafish calcium imaging in Ref. 152, scale bar: 100  μm. (b) The FLFM family is presented with MLA and is placed at the Fourier plane (upper) formed by FL (Fourier lens). The XLFM (middle) has a variation focal length MLA to perform DOF extension. Confocal LFM (bottom) applied the laser scanning and filtering the emission by a mask placed right after MLA. One neuroimaging example in Drosophila from Ref. 153, scale bar: 100  μm. (c) Scanning LFM family with DAOSLIMIT LFM or sLFM includes a 2D-galvo scanner and csLFM with a piezoelectric tip with a tilt platform for increased spatial sampling rates. In csLFM, the synchronization between line scans and the camera rolling shutter mode at the emission path of the microscopy can reduce the out-of-focus by illuminating the specific region and forming a virtual slit. Both of them showed a potential in neuro recording in complex scattering samples such as awake mouse brains, whereas csLFM gave a clearer image with better SBR. Result from Ref. 154, scale bar: 100  μm. The images of neurons are reproduced and adapted with permission from Refs. 152–154, respectively.

Before discussing LFM, it is essential to briefly introduce the principle of the “light field.” In space, light rays are distributed in all directions as vectors. A light field is a collection of these vector distributions, represented by a five-dimensional plenoptic function (5DPF), as shown in Fig. 3(a) (left). Due to its computational complexity, the 5DPF is often simplified to a 4D representation in space and includes four parameters: u, v, s, and t. The details are explained elsewhere.^155^

To achieve 4D multiplexing, a microlens array (MLA) is introduced into the system, which is the key component that makes LFM unique. Conventionally, the MLA is placed on the intermediate image plane.^156^ When choosing an MLA, it is important to match the F-number (F#) of the MLA with the F-number of the objective lens,^157^^,^^158^ as shown by the equation from Ref. 159

$$F\#=\frac{f_{\mathrm{MLA}}}{d_{\mathrm{MLA}}}=\frac{M_{\mathrm{obj}}}{2\mathrm{NA}},$$

where $f_{\mathrm{MLA}}$ and $d_{\mathrm{MLA}}$ are the focal length and pitch size of MLA, respectively, and $M_{\mathrm{obj}}$ and NA are the magnification and numerical aperture of the objective lens, respectively. In practice, this F#-matching should be as close as possible to prevent overlapping and separation between the lenslet patterns on the image. A relay lens, placed between the tube lens and MLA, can be considered a solution for F# matching. Using a relay,^160^ the magnification is shown below, which can provide better alignment between the MLA and the objective lens

$$M_{\text{relay}}\approx \frac{2f_{\mathrm{MLA}}/d_{\mathrm{MLA}}}{M_{\mathrm{obj}}/\mathrm{NA}}.$$

Building on the concept of the “light field,” Ref. 150 implemented the MLA at the image plane of the microscope to create conventional LFM. However, commercial MLAs often have short focal lengths, which can make the physical establishment of LFM challenging. To address this, a relay lens can be used to conjugate the image onto the camera sensor. Although a 4f-system relay lens is an option, using a macrolens can offer a range of magnifications (if needed) and help reduce aberrations around the edges of the image.^152^ Currently, several types of LFM have been introduced with a wide range of biological applications. The following information will focus specifically on the LFM development for *in vivo* neuroimaging.

The raw light field image is initially reconstructed using the light ray model. Although this model potentially works well for light field cameras, it is less effective for microscopy due to issues related to diffraction and resolution.^151^ In 2013, the wave optics theory with 3D deconvolution was introduced for LFM,^161^ offering an approach for volume reconstruction with improved lateral resolution. This method was applied to zebrafish whole-brain calcium imaging^152^ [Fig. 3(a), right], inspiring numerous LFM studies. However, the point-spread-function (PSF) values vary throughout the volume, leading to high computational costs.^151^

To address these issues, Fourier light field microscopy (FLFM)^162^ was developed. A Fourier lens is placed between the tube lens and the MLA [Fig. 3(b), top left], shifting the MLA to the Fourier plane. This reduces artifacts and improves volume reconstruction results. As FLFM divides collected information into multiple sections, the field-of-view (FOV), depth-of-field (DOF), and resolution are no longer interdependent.^163^ Several solutions have been proposed to optimize these parameters. For instance, XLFM (extended-FOV LFM) uses a customized MLA with varying focal lengths to optimize resolution and increase DOF through multifocal imaging [Fig. 3(b), middle left].^164^

In 2020, the sparse decomposition algorithm was introduced,^153^ enhancing resolution by evenly reconstructing both dense and sparse information. This technique was applied to Drosophila calcium imaging [Fig. 3(b), right]. Confocal LFM reduces background noise by placing a mask right after the MLA, similar to the pinhole in traditional confocal microscopy, significantly improving DOF and resolution [Fig. 3(b), bottom left].^165^

Another technique achieving 220-nm lateral resolution and 400-nm axial resolution involves 2D scanning in the emission path, known as digital adaptive optics scanning light field mutual iterative tomography (DAOSLIMIT), or scanning LFM (sLFM)^166^ [Fig. 3(c), left]. sLFM achieves a large FOV and high spatiotemporal resolution through aberration corrections in various biological samples with low phototoxicity, although it has limited depth. Instead of using a 2D-galvo scanner, confocal scanning LFM (csLFM) employs a piezoelectric tip and tilt platform for scanning in the emission path [Fig. 3(c)], improving the signal-to-background ratio (SBR). This system csLFM synchronizes the line scanning illumination with the rolling shutter of the camera, achieving near-diffraction-limit resolution by selectively focusing on specific volume and reducing out-of-focus signals and background noise.^154^ This setup functions as a confocal microscopy with a virtual slit instead of a physical pinhole.

**Fig. 4:**
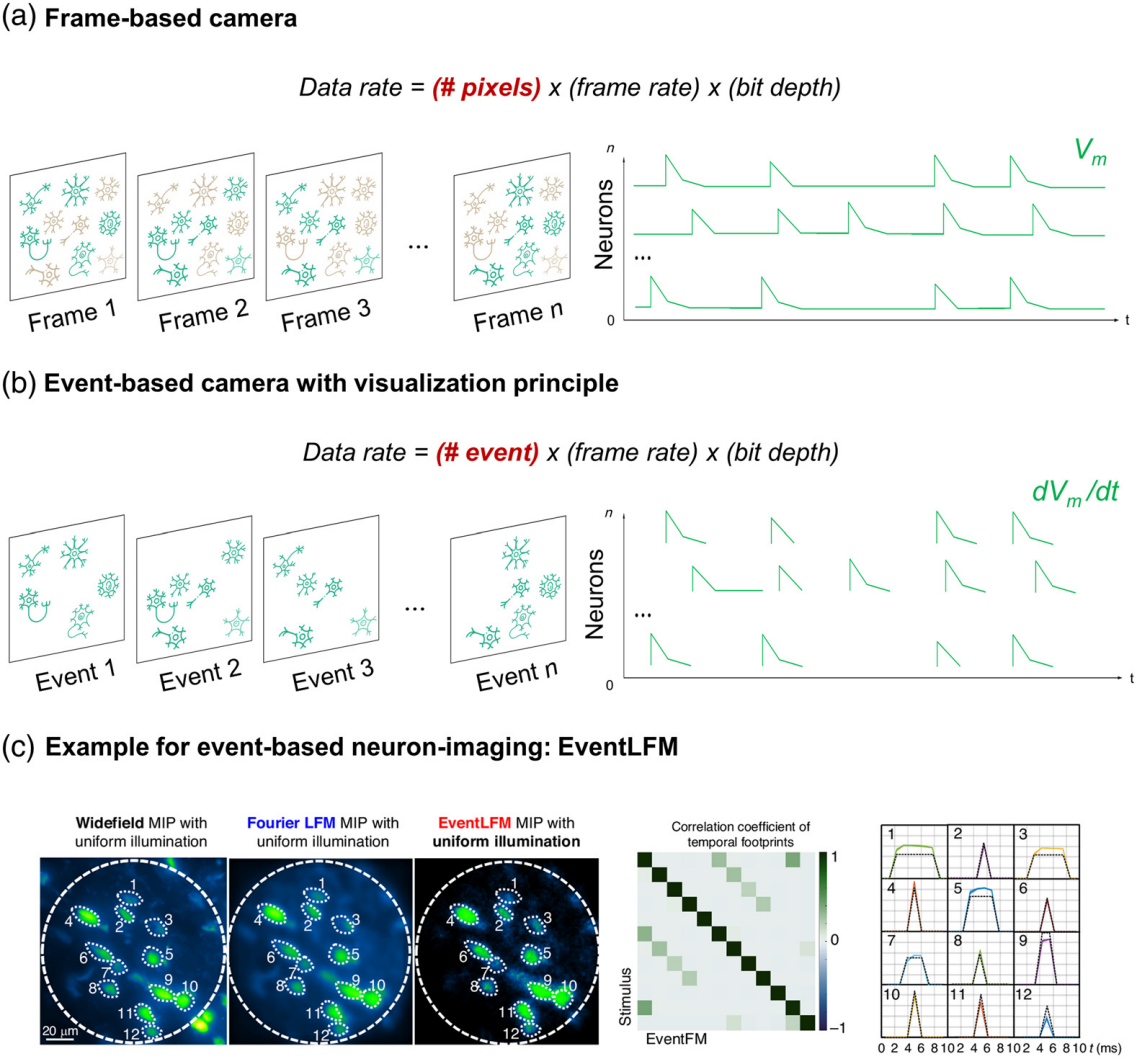
Principles comparison between frame-based camera and event-based camera: (a) Frame-based cameras capture neuronal activity in discrete frames at set time intervals, producing a sequence of images. This approach captures the entire scene per frame, which can lead to redundant data for static elements and motion blur for rapidly moving objects due to insufficient temporal resolution. (b) Event-based cameras, by contrast, capture changes asynchronously by recording “events” only when there is a change in brightness, either an “on” or “off,” then reduces data redundancy and enhances temporal resolution, ideal for capturing rapid neuronal firing dynamics. (c) Application of event-based imaging in neuroimaging: EventLFM combines the high speed of event cameras with the 4D imaging capabilities of LFM to record neuronal activity in mouse brain tissue. The performance of EventLFM with widefield imaging and Fourier LFM shows improved correlation of neuronal traces with stimuli and reduced data redundancy. The normalized cross-correlation between EventLFM neuron traces and sorted stimuli demonstrates the method’s precision in capturing rapid neuronal responses, with averaged intensity and standard error for each neuron’s spike train closely matching the intensity-normalized stimulus response. Adapted with permission from Ref. 167.

Both sLFM and csLFM have demonstrated high performance in LFM studies, particularly in overcoming the limitation of LFM in highly scattered samples such as mouse brains, successfully tracking neuronal activity [Fig. 3(c)]. A hybrid technique called EventLFM combines the power of FLFM with kHz recording capability using an event camera for neuronal signals in mouse brain tissue [Fig. 4(c)] and applying it to moving *Caenorhabditis elegans*.^167^ Squeezed LFM overcomes LFM’s resolution limitations by introducing dual-light sheet illumination for high-contrast imaging, achieving up to kilohertz volume rates in red blood cell flow recording (1000 volumes per second) and voltage imaging in leech ganglions (800 volumes per second).^168^

As previously mentioned, LFM is a technique for capturing information in the form of a “light field,” which contains 3D volume information and requires a reconstruction process.^169^ Initially, the ray-optics model, which sums rays from different angular views, was applied to reconstruct the LFM data.^150^^,^^170^^,^^171^ Detailed equations and explanations of this model can be found.^169^^,^^170^ Although this method provided rapid reconstruction, it resulted in low resolution.^169^

To address this, a reconstruction method based on the wave-optics model combined with deconvolution was introduced.^161^ This method involves deconvolving the system’s PSF and applying the Richardson–Lucy deconvolution with appropriate iterations, providing a standard reconstruction method for conventional LFM.^152^ However, the system’s PSF is spatially complicated, resulting in high computational cost. However, the spatial complexity of the system’s PSF leads to high computational costs. FLFM reconstruction presents a promising method to overcome some of these limitations using an invariant PSF. However, as previously mentioned, the process of splitting information in FLFM can make it challenging to balance different image properties.

The current trend in computer science is the application of deep learning techniques. Unlike traditional microscopy, where the input is a low-quality image and the output is a high-quality image of the same dimensionality, LFM data require both reconstruction (converting low-quality 2D images with depth information) and enhancement of image quality (resulting in high-quality 3D image stacks). Most deep learning approaches for light field imaging involve both LFM and general images generated from other types of microscopy.

In 2021, a combination of LSM and LFM, along with a volume reconstruction deep learning method based on a convolutional neural network (CNN) model, was applied to neuronal calcium activity imaging.^172^ In the same year, a wave-optics-based deep learning method called view-channel-depth neural network (VCD-Net) was introduced, pairing high-resolution confocal images with light field images for network training.^173^ VCD-Net has been widely used in various applications, including movement tracking of *C. elegans* in microfluidic chips,^174^ and in myocardium imaging when combined with aberration modeling and light field techniques.^175^ VCD-Net has since been upgraded to VCD-2.0, offering improvements in both resolution and speed.^176^

Another approach, known as the iterative shrinkage-thresholding algorithm, where each iteration corresponds to a network layer, demonstrated effective performance in calcium neuron imaging by combining training data from two-photon and light field images.^177^ A physics-based framework named virtual-scanning LFM^178^ enhanced DAOSLIMIT-LFM, improving resolution for *in vivo* applications such as heartbeat imaging in embryonic zebrafish, voltage imaging in *Drosophila*, and mouse liver imaging.

Most recently, in 2024, the convolutional iterative shrinkage and thresholding algorithm (CISTA-net)^179^ was introduced. It obtained ground-truth data from both two-photon and wide-field imaging, alongside a large set of LFM images at different depths. CISTA-net was applied to neuron activity imaging and has the potential to inspire further developments in neuroimaging techniques.

With the diverse configurations and processing methods available, LFM has demonstrated significant potential for neuroimaging. Various animal models, including *C. elegans*,^152^^,^^174^ zebrafish,^152^^,^^165^^,^^166^^,^^180^ and *Drosophila*^153^^,^^166^ have been employed for neuronal recording. Although most applications focus on these simpler animal models due to the challenges of light scattering in mammalian tissues, some studies have shown promise for neuroimaging in the mouse brain.^165^^,^^166^^,^^180^^–^^182^ Table 3 summarizes the neuroimaging applications in the light field strategy.

**Table 3:** Neuroimaging application of some LFM methods.

Samples	LFM detection	LFM illumination	Volume (μm)	Resolution (μm)	Reconstruction processing	Reference
*C. elegans*	Conventional LFM	Wide-field illumination	350 × 350 × 30 (5 and 50 Hz)	Lateral: ∼1.4	Wave-optics	152 ^a^
				Axial: ∼2.6		
			590 × 590 × 31 (33 Hz)	Lateral: ∼1.9	VCD-Net (microfluidic chip for ground truth)	174
				Axial: ∼2.0		
Zebrafish	Conventional LFM	Wide-field illumination	700 × 700 × 200 (20 Hz)	Lateral: ∼3.4	Wave-optics	152
				Axial: ∼11		
			700 × 700 × 180 (24 Hz)	Lateral: ∼1.8	3D deconvolution with a multiscale scattering model	180
				Axial: ∼2.5		
		Speckle illumination	170 (depth) (10 Hz)	1.4 times higher compared with conventional LFM	Wave-optics	182
	FLFM (confocal LFM)	Selective illumination	ø800 × 200 (30 Hz)	Lateral: ∼2.1	Frame-by-frame unbiased	165 ^a^
				Axial: ∼2.5		
	Scanning LFM	Wide-field illumination	225 × 225 × 16 (30 to 100 Hz)	Lateral: ∼0.22	Aberration-corrected 3D reconstruction	166
				Axial: ∼0.40		
		Line-scanning with a rolling shutter	100 (depth)	Lateral: ∼0.3		154 ^a^
				Axial: ∼1.0		
Drosophila	FLFM (XLFM)	Altered between wide-field and light sheet illumination	700 × 400 × 300 (2 to 50 Hz)	Lateral: ∼3.5	Sparse decomposition and Richardson–Lucy iterations	153
				Axial: ∼7.4		
	Scanning LFM	Wide-field illumination	225 × 225 × 16 (30 to 100 Hz)	Lateral: ∼0.22	Aberration-corrected 3D reconstruction	166
				Axial: ∼0.40		
		Line-scanning with a rolling shutter	100 (depth)	Lateral: ∼0.3		154 ^a^
				Axial: ∼1.0		
Mouse	FLFM (confocal LFM)	Selective illumination	ø800 × 10 (70 Hz)	Lateral: ∼4.0	Frame-by-frame unbiased	165 ^a^
				Axial: ∼6.4		
	Conventional LFM	Speckle illumination	170 (depth) (10 Hz)	1.4 times higher compared with conventional LFM	Wave-optics	182
		Wide-field illumination	700 × 700 × 180 (24 Hz)	Lateral: ∼1.8	3D deconvolution with a multiscale scattering model	180
				Axial: ∼2.5		
		Head-mounted miniscope	700 × 600 × 360 (16 Hz)	Lateral: ∼6.0	Seeded iterative demixing	181
				Axial: ∼30		
	Scanning LFM	Wide-field illumination	225 × 225 × 16 (30 to 100 Hz)	Lateral: ∼0.22	Aberration-corrected 3D reconstruction	166
				Axial: ∼0.40		
		Line-scanning with a rolling shutter	150 (depth)	Lateral: ∼0.3		154 ^a^
				Axial: ∼1.0		

a The experiments used different objective lens.

LFM captures volumetric information in a single exposure, offering rapid 3D or 4D imaging of transient events. However, dividing the sensor with a microlens array reduces per‐voxel signal, leading to lower spatial resolution—often 3 to 5× worse than a comparable microscope without an array.^183^^–^^185^ Likewise, although modern sCMOS cameras used in LFM can achieve quantum efficiencies upwards of 80%, certain scanning microscopies employ single‐photon detectors that excel under low‐light conditions. Consequently, LFM does not necessarily improve photobleaching or signal‐to‐noise relative to scanning techniques, which can dwell on each voxel to accumulate more photons (though at the cost of speed). Overall, the choice between LFM and scanning microscopy should be guided by the experimental priority—whether faster volumetric capture or higher spatial resolution and SNR—and the availability of high‐efficiency detector technology.

### 3.3 Event-Based Strategy: From Frame to Event

Considering that a fundamental aspect of neuronal studies is visualization, voltage imaging holds great promise for neuronal imaging. Ultrafast techniques must adapt to the demands of voltage imaging by incorporating high-speed mechanisms, such as kilohertz MPM. Although microscopy techniques can achieve imaging speeds of kHz, the acquisition rate of imaging sensors typically remains around 100 fps.^186^

The development of scientific cameras has focused on improving sensitivity, speed, and image quality. The invention of the charged-coupled device (CCD) in 1969 revolutionized scientific imaging. Over the following decades, CCD technology was continuously updated in terms of pixel resolution, dynamic range, and readout speeds, making it the dominant choice for scientific cameras during that ear. The introduction of electron multiplying CCD (EMCCD) or “on-chip multiplication” further enhanced sensitivity using electron multiplication to amplify the signal, resulting in better performance under low-light conditions compared with traditional CCDs.

The next significant advancement came with the development of complementary metal oxide semiconductor (CMOS) technology, which uses complementary pairing of nMOS and pMOS transistors as logical switches to form a digital circuit, in contrast to the analog nature of CCDs. This led to the emergence of scientific CMOS (sCMOS), which offers a good balance among sensitivity, dynamic range, and speed, making it particularly suitable for capturing fast biological processes, including voltage imaging, as demonstrated in recent studies.^107^^,^^187^^–^^190^ Although CCD, EMCCD, and sCMOS technologies have shown potential in bioresearch, they still face challenges related to high noise levels. A CMOS emerging technology called photon-number resolving^191^ aims to surpass both CCD and sCMOS in terms of sensitivity and noise performance. This technology is capable of accurately measuring photon numbers with low readout noise, providing more precise measurements.^192^ The comparison of each camera type is shown in Table 4.

Although traditional and the latest detectors offer many advantages, understanding neurons necessitates millisecond-level frame measurements. These cameras typically use frame-based recording techniques [Fig. 4(a)], capturing frames at fixed intervals and recording the entire scene at once, which can result in redundant data for static parts of the scene. A new camera technology, called the event camera (EC)^193^ (also known as dynamic vision sensor or neuromorphic camera), addresses this issue by recording “event generation” or changes in brightness as positive/ON (increased brightness) and negative/OFF (decreased brightness) [Fig. 4(b)].

Each pixel in an event camera operates independently and asynchronously, detecting changes in light intensity rather than capturing full frames at regular intervals. The recorded data include pixel position (x,y), occurrence time (t), and polarity (p).^193^ Event cameras generate data only when changes occur, allowing for more precise capture of fast movements.^194^^,^^195^ Because of this property, event cameras transmit data as soon as changes are detected. This results in minimal latency, often less than one millisecond, which is crucial for real-time applications.^194^^,^^195^ Event cameras can handle a very wide range of lighting conditions, from very dark to very bright scenes, without losing detail. Their dynamic range often exceeds 120 dB, far superior to the 60 dB typically seen in high-quality frame-based cameras.^193^^,^^196^ Due to the independent operation of each pixel, ECs can detect changes with high speed and responsiveness,^193^^,^^195^^,^^197^^,^^198^ achieving time resolutions equivalent to 10,000 fps.

Based on the principle of EC, strategies for illumination should be carefully considered. LEDs and lasers are widely used as light sources in traditional microscopy for imaging applications. For event imaging, precise control of the illumination period can be employed to manage the ON/OFF states, such as using an electronic chopper or an LED in pulse mode. Although EC offers high temporal resolution, advancing these applications requires improvements in spatial resolution. By integrating single-molecule localization imaging,^197^ which has been proven to overcome the diffraction limit, high spatio-temporal resolution techniques have been developed, enabling event-localization imaging.

Event-based imaging methods have also proven applicable to biological samples. For instance, Basumatary et al.^195^ demonstrated successful imaging of mitochondrial membrane proteins and cells. Zhang et al.^196^ applied event-based methods for cell counting and size calculation, also comparing results between EC and sCMOS. Other researchers have imaged live cells and compared results with sCMOS and EMCCD.^197^ EventLFM, a hybrid technique combining LFM with EC, uses targeted illumination via a digital micromirror device (DMD) to selectively illuminate neurons [Fig. 4(c)] and reduce background noise. The DMD also generated the switching on/off for neuron capture. Deep learning can be applied to EC, such as with the DeepTrack algorithm,^194^ which was used to analyze the ON/OFF information from the EC. As mentioned earlier, EventLFM combines the ultrafast capabilities of LFM and EC, achieving resolutions similar to those of FLFM.

**Table 4:** Comparison of event-based camera and frame-based camera.

Types	Event camera (EC)	Scientific CMOS (sCMOS)^a^	Photon quantification camera^a^
Principle	Asynchronous, event-driven	Frame-based, digital operation	Similar to sCMOS but can count photons
Pixel size (μm)	∼4.86	∼4.6 to 6.5	∼4.6
Dynamic range (dB)	∼86 to 120	∼90	∼87
Bit depth	1	8/12/16	8/12/16
Frame rate (fps, full resolution)	∼10,000 (time resolution equivalent)	∼100	∼115
Pixel fill factor (%)	22 to 77	60 to 70	60 to 70
Quantum efficiency	∼7.4%	∼86% (peak QE)	∼85% (peak QE)
Readout noise	Lower readout noise with minimal dark current noise occasionally causing “false events”	1.0 electrons (rms)	030 to 0.43 electrons (rms)
		0.9 electrons (median)	0.25 to 0.39 electrons (median)
Dark current		0.6 electrons/pixel/s	0.006 electrons/pixels/s
Readout speed	No defined readout speed due to their asynchronous operation	∼115 frame/s	∼120 frame/s
Data rate/readout throughput (only for EC)	20 Mevents/s – 50 Mevents/s	∼1.4 Gbit/s	∼1.4 Gbit/s

a It is the average number; the exact number is based on specific cameras.

To summarize, event cameras (ECs), with their unique asynchronous operation and event-driven data capture, offer several significant advantages over traditional frame-based cameras, particularly for biological applications. ECs provide exceptional temporal resolution, making them well-suited for visualizing and detecting fast events. However, processing and interpreting the asynchronous, event-driven output requires specialized algorithms, which can complicate software development and implementation. In addition, external support, such as DMD as used in Ref. 167, is often necessary to create on/off levels for observing neuronal signals. Although ECs excel in temporal resolution, their spatial resolution is lower compared with high-end traditional cameras such as sCMOS or qCMOS, which can limit their application in detailed recognition tasks.

## 4 Conclusion

Each ultrafast optical imaging technology discussed in this review presents distinct advantages while also facing certain limitations. KHz-2PM excels in delivering high temporal resolution for deep tissue imaging but is constrained by photodamage risks and system complexity. LFM enables rapid 4D imaging with potential applications in real-time volumetric imaging, although it may sacrifice spatial resolution to enhance temporal resolution. Event-based imaging provides unparalleled temporal resolution but encounters challenges in reconstructing static scenes and requires advanced computational techniques for data interpretation.

The integration of these complementary technologies could significantly enhance overall imaging capabilities, leading to a more comprehensive understanding of neuronal dynamics. By leveraging the strengths of each technique, such as combining the deep tissue penetration and high resolution of kHz-2PM with the real-time volumetric imaging capabilities of LFM, it is possible to overcome individual limitations.

The evolution of ultrafast optical imaging technologies is poised to drive future breakthroughs not only in neuroscience but also across various medical and scientific fields. In clinical diagnostics, these technologies could lead to earlier and more accurate detection of neurological disorders by enabling the visualization of disease progression at the cellular level. In drug discovery and development, ultrafast imaging could facilitate high-throughput screening of neuroactive compounds, accelerating the identification of potential therapeutic agents. In addition, these techniques could provide new insights into brain function and plasticity, enhancing our understanding of learning, memory, and behavior.

## Data Availability

As this is a review paper, no original code or data were generated or analyzed. Therefore, a Code and Data Availability statement is not applicable.
